# Cell Sheets from Adipose Tissue MSC Induce Healing of Pressure Ulcer and Prevent Fibrosis via Trigger Effects on Granulation Tissue Growth and Vascularization

**DOI:** 10.3390/ijms21155567

**Published:** 2020-08-04

**Authors:** Natalya Alexandrushkina, Peter Nimiritsky, Roman Eremichev, Vladimir Popov, Mikhail Arbatskiy, Natalia Danilova, Pavel Malkov, Zhanna Akopyan, Vsevolod Tkachuk, Pavel Makarevich

**Affiliations:** 1 Medical Research and Education Center, Lomonosov Moscow State University, Lomonosovskiy av., 27-10, 119191 Moscow, Russia; nimiritsky@gmail.com (P.N.); romaneremichev@gmail.com (R.E.); natalyadanilova@gmail.com (N.D.); malkovp@gmail.com (P.M.); zhanna.fbm@gmail.com (Z.A.); vtkachuk@mc.msu.ru (V.T.); pmakarevich@mc.msu.ru (P.M.); 2 Faculty of Medicine, Lomonosov Moscow State University, Lomonosovskiy av., 27-1, 119192 Moscow, Russia; galiantus@gmail.com (V.P.); homomedicus@mail.ru (M.A.)

**Keywords:** cell sheet, mesenchymal stromal cells, wound healing, granulation tissue, angiogenesis, endothelial cells, vessel stabilization, skin regeneration, pressure ulcer

## Abstract

We report a comparative study of multipotent mesenchymal stromal cells (MSC) delivered by injection, MSC-based cell sheets (CS) or MSC secretome to induce healing of cutaneous pressure ulcer in C57Bl/6 mice. We found that transplantation of CS from adipose-derived MSC resulted in reduction of fibrosis and recovery of skin structure with its appendages (hair and cutaneous glands). Despite short retention of CS on ulcer surface (3–7 days) it induced profound changes in granulation tissue (GT) structure, increasing its thickness and altering vascularization pattern with reduced blood vessel density and increased maturation of blood vessels. Comparable effects on GT vascularization were induced by MSC secretome, yet this treatment has failed to induce repair of skin with its appendages we observed in the CS group. Study of secretome components produced by MSC in monolayer or sheets revealed that CS produce more factors involved in pericyte chemotaxis and blood vessel maturation (PDGF-BB, HGF, G-CSF) but not sprouting inducer (VEGF165). Analysis of transcriptome using RNA sequencing and Gene Ontology mapping found in CS upregulation of proteins responsible for collagen binding and GT maturation as well as fatty acid metabolism enzymes known to be negative regulators of blood vessel sprouting. At the same time, downregulated transcripts were enriched by factors activating capillary growth, suggesting that in MSC sheets paracrine activity may shift towards matrix remodeling and maturation of vasculature, but not activation of blood vessel sprouting. We proposed a putative paracrine trigger mechanism potentially rendering an impact on GT vascularization and remodeling. Our results suggest that within sheets, MSC may change their functional state and spectrum of soluble factors that influence tissue repair and induce more effective skin healing inclining towards regeneration and reduced scarring.

## 1. Introduction

Healing of damaged tissue is a dynamic process regulated by cytokines and growth factors, proteolytic enzymes and extracellular matrix (ECM). They serve as regulatory cues for cells and tissue structures, including blood vessels, nerves etc. Different stages wound healing involve blood cells (platelets and immune cells), stromal cells (myo/fibroblasts, endothelium, etc.), parenchymal and stem/progenitor cells [1]. After phases of hemostasis and inflammation, healing turns to proliferative stage, resulting in formation of a provisional structure known as granulation tissue (GT) that substitutes primary blood clot disrupted by fibrinolytic enzymes. GT in humans is rich with blood vessels embedded in ECM filled by activated stromal and immune cells. Later GT structure changes dramatically due to a process termed *remodeling*, which results in either formation of pre-existing tissue (regeneration) or a non-functional scar at the site of damage (fibrosis), typically accompanied by hypertrophy/hyperplasia of remaining tissue to retain organ function. Wound healing interruption at any stage or ablation of its critical participants may result in non-healing wounds, excessive scarring or subsequent neoplasms [2].

In cutaneous wounds of different natures (burns, trophic ulcers, radiation injury), acceleration of closure is an important point as far as it prevents infection of the wound bed and its potential life-threatening complications. Another crucial endpoint is the limitation of scarring—a naturally developed response to deep or vast injury of skin [3]. After scar formation, growth of skin appendages (hair, glands, fingernails) or recovery of dermal layers is blocked. Basically, in most tissues, a scar forms a “non-receptive” site for both endogenous regeneration and regenerative medicine interventions [4].

Thus, in cutaneous injury, a therapeutic approach that can stimulate rapid defect closure and reduce fibrosis and potentially provides ground for more effective regeneration of skin and its appendages. In recent decades, promising methods using multipotent mesenchymal stromal cells (MSC) from different sources acquired significant attention and addressed skin-healing issues [5]. Feasible isolation and culture along with active secretion of ECM, cytokines and growth factors made MSC a valuable “medicinal stromal cell” for cutaneous repair [6,7]. Furthermore, composition of MSC secretome that mediates angiogenic, immunomodulating, antiapoptotic and proliferative effects of MSC allowed its independent application for “cell therapy without cells” [8,9].

Finally, the ability of MSC to form tissue-like constructs composing of viable cells and their native ECM resulted in the development of cell sheets (CS)—minimal tissue-engineered constructs that found application for many conditions. Our previous experience with MSC-based CS showed their efficacy for stimulation of angiogenesis, nerve repair and cardiac regeneration and proved their superiority over routinely used local injection [10,11,12,13,14]. The latter has been shown to induce MSC apoptosis and its rapid clearance from blood flow or tissue (muscle, myocardium, etc.). To our surprise, we found few data about the efficacy of MSC-based CS for treatment of pressure ulcers and no studies implementing direct comparison of CS vs. secretome from MSC.

Pressure-induced lesions are a widely spread condition that remains a clinical problem with few cell-based products for its treatment. Due to the specific nature of damage that induces pressure, published studies on ulceration using surgical wound models can hardly be considered relevant evidence, and filling this gap became a practical rationale for the present study.

We were also intrigued by changes in secretome and transcriptome of MSC that may occur within CS, which is believed to present a more “tissue-like” environment for cells [15,16]. However, the degree of cell status change after CS assembly remains poorly investigated. Certain works in spheroids or cell-seeded scaffolds included RNA sequencing to show transcriptome profile including the signature of reprogramming/dedifferentiation in MSC. Modality of transcriptomic changes in CS is marginally unexplored besides certain studies that used PCR for specific groups of genes or regulatory RNAs [17,18,19,20].

Our work was intended to provide data expanding our view on CS as a tissue-engineered construct for acceleration of cutaneous defect healing and addresses the pressure ulcer issue. Going beyond applied task of MSC-based therapies we obtained data on transcriptome and secretome changes to illustrate the shift in MSC status induced by formation of CS.

## 2. Results

### 2.1. MSC Sheet Transplantation Accelerates Healing of Pressure Ulcer Defect

**Figure 1:**
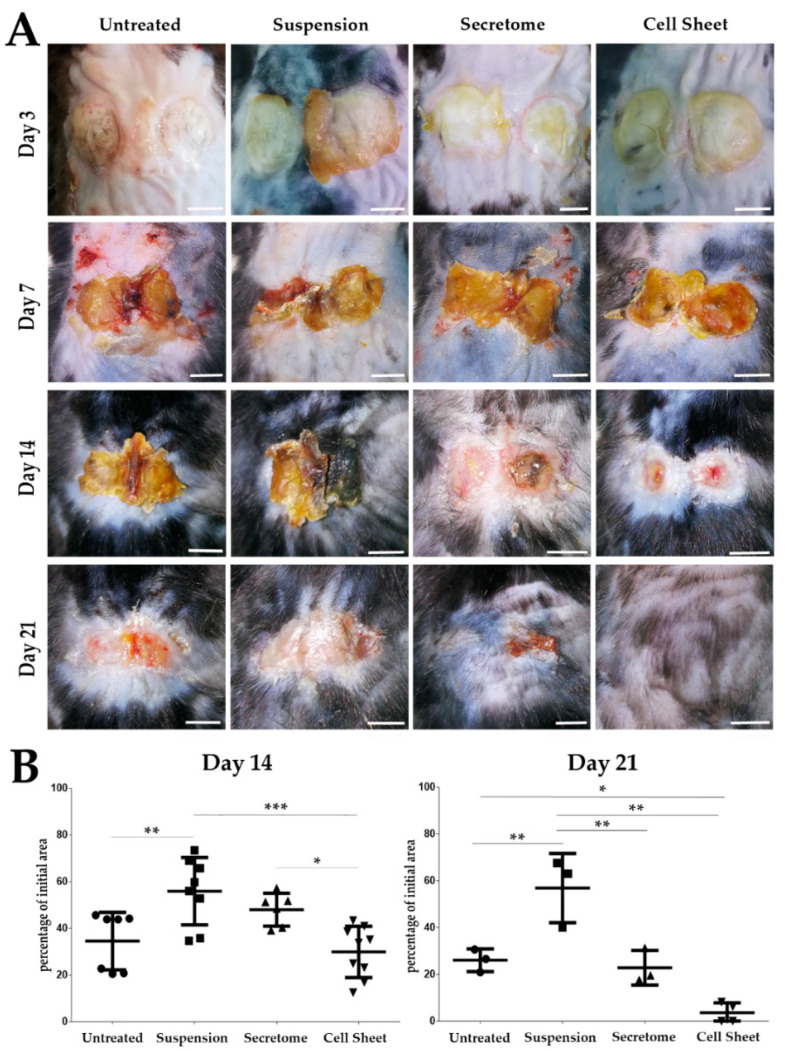
Macroscopic evaluation of pressure ulcer closure in experimental groups. (**A**) At Day 21 after cell sheet transplantation we found complete closure of the defect and onset of hair growth at the site of treated pressure ulcer. Scale bar represents 5 mm. (**B**) Results of defect relative area measurement as % of initial pressure ulcer at Days 14 and 21. Days 3 and 7 showed no significant difference between groups (*—*p* < 0.05; **—*p* < 0.01; ***—*p* < 0.001).

Dynamics of ulcer closure were calculated as relative change (%) of defect surface area normalized to initial defect in photos taken at Days 3, 7, 14 and 21 of the experiment. No difference between groups was found at days 3 and 7; however, starting from Day 14, acceleration of ulcer closure was observed in CS application group (“Cell Sheet”) (Figure 1A). By Day 14, area of defect in this group decreased to one-third of the initial 1.2 cm^2^ size and was significantly lower (Figure 1B, left plot) than in animals treated by injection of suspended MSC (“Suspension”) or MSC secretome (“Secretome”). At the same time, we found no difference between “Cell Sheet” and “Untreated” negative control at Day 14. At Day 21 relative ulcer area in the “Cell Sheet” group was significantly lower than in untreated control and “Suspension” group, while the difference between “Cell Sheet” and “Secretome” groups (Figure 1B, right plot) did not reach statistical significance (*p* = 0.063). However, only in the “Cell Sheet” group did we find animals (3/4 mice followed up by Day 21) with hair growth indicating partial recovery of skin appendages (Figure 1A). This observation was supported by histology study (Figure S1) and detailed analysis of individual animal specimen (Supplementary Material M1) that displayed the most prominent healing of dermis in the “Cell Sheet” group.

Evaluation of ulcer closure in “Suspension” group showed that injection of MSC surprisingly slowed this process compared to untreated control at Day 14 (57.8 ± 14.4% vs. 43.8 ± 12.3% area respectively; *p* = 0.0082) and Day 21 (63.0 ± 14.8% vs. 26.6 ± 4.8% area respectively; *p* = 0.0071). MSC secretome injections yielded results comparable with untreated control throughout the experiment, not reaching statistical significance at endpoint (Figure 1B, right plot).

### 2.2. Cell Sheet Transplantation Induces Intensive Remodeling of Granulation Tissue

**Figure 2:**
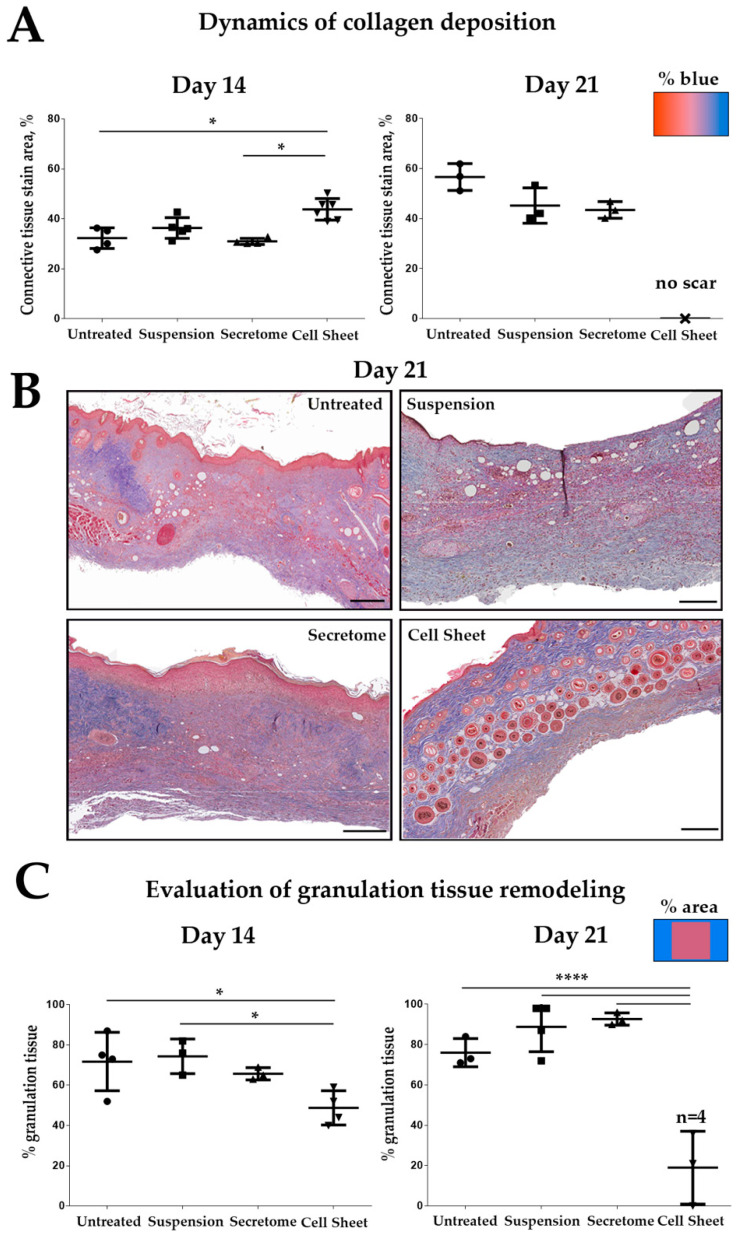
Histological assessment of granulation tissue (GT) remodeling in experimental groups. (**A**) Relative content of collagen and reticular fibers (% of defect); (**B**) healing of skin and its appendages at the site of pressure ulcer in the “Cell Sheet” group (Day 21); Masson’s trichrome, scale bar represents 200 µm; (**C**) assessment of granulation tissue remodeling by calculation of its relative area (% of section area); *n* = 4 denotes number of animals assessed at this point, of which 3 had no GT area to account for while 1 had minimal remaining GT which allow presenting statistics (*—*p* < 0.05; ****—*p* < 0.0001).

We assessed the process of GT remodeling using a three-color Masson staining to visualize connective tissue ECM deposition (Figure 2). Remodeling results in reduction of GT mass, apoptosis of fibroblasts and vessel regression followed by deposition and thickening of collagen bundles. At Day 14 we found significantly increased amount of collagen and reduction of GT area in sections from the “Cell Sheet” group vs. the untreated control (Figure 2—Day 14), while “Suspension” and “Secretome” groups showed results similar to untreated control.

At Day 21 “Cell Sheet” group showed a dramatic drop of GT area with obvious dermis healing (Figure 2—Day 21) supporting previous macroscopic assessment (Figure 1A). Indeed, hair follicles and dermal glands were found only in histological sections from “Cell Sheet” group animals (Figure 2B, Figure S1 and Supplementary Material M1). To ensure that complete healing was not a feature of the animal model we performed additional evaluation at late term (Day 35) in several untreated mice. We found that once followed-up till Day 35, untreated animals show scarring at the site of the defect (Figure S2). Thus, transplantation of CS did not result in acceleration of inevitable healing but changed the outcome preventing fibrosis.

It should be noted that at Day 21, skin samples from “Cell Sheet” group showed the presence of blue dye, indicating connective tissue deposition, yet this pattern in Masson stain is typical for healthy skin rich with collagens (Figure S3). The presence of skin appendages within area of healing supports that this connective tissue is not related to scarring but to dermal ECM visualized by the same bluish stain as in fibrosis.

### 2.3. Evaluation of Transplant Retention after MSC Delivery by Suspension or Cell Sheets

**Figure 3:**
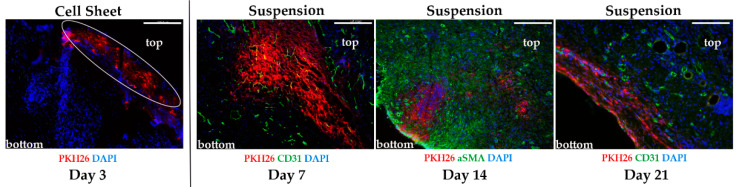
Retention of mesenchymal stromal cells (MSC) labeled by PKH26 after transplantation to pressure ulcers. Cell sheet (CS) is detected on wound surface only at Day 3; PKH26 signal from injected MSC is detectable in the tissue up to Day 21; “top” marks location of wound bed, “bottom” marks deeper layers of skin. Immunofluorescence, scale bar represents 250 µm.

We pre-labeled suspended MSC and CS by PKH26 fluorescent dye and transplanted them to experimental animals with pressure ulcers. To our surprise after injection of pre-stained MSC PKH26 signal was visualized in dermis and subcutaneous layers at Day 21 of the experiment (Figure 3). Indeed, in the “Suspension” group, we found PKH26-positive cells localized between αSMA-positive myofibroblasts (Figure 3, Day 14) or adjacent to CD31-positive blood vessels (Figure 3, Day 21).

Signal from labeled CS was detected only in the first evaluation point—Day 3. In certain images from the “Cell Sheet” group, PKH26 was not co-localized with DAPI-positive nuclei, which may hint at a residual nature of PKH26 signal due to its leakage during early apoptosis of MSC. We failed to detect PKH26-positive CS in any specimen taken at Day 7 (*n* = 4), which suggested early rejection of CS along with scab by natural detachment of its mass or during removal of wound dressing. Assessment of secretome retention by this method was not possible, but published studies describe the half-life of delivered active fraction ranging from several hours to 1–2 days [21,22].

### 2.4. Cell Sheets Promote Formation of Granulation Tissue at Early Stages of Healing

**Figure 4:**
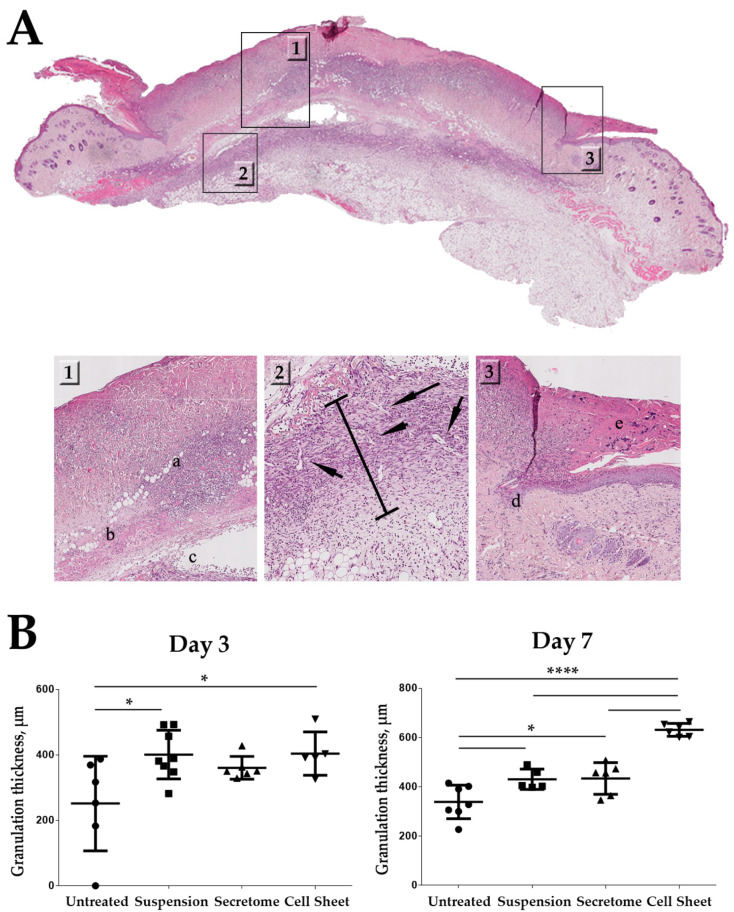
Influence of cell sheets, suspended MSC or MSC secretome on GT thickness in healing pressure ulcer. (**A**) Representative image of a histological section at Day 3; panel markup: **1**—zone of damaged tissue down to (**a**) muscular layer with (**b**) immune cells infiltration and (**c**) edema of underlying tissue; **2**—zone of GT formation; bar indicates measured thickness, arrows indicate blood vessels; **3**—defect surface with (**e**) scab and (**d**) margin of migrating epithelial layer covering ulcer. Hematoxylin-eosin staining, scale bar represents 50 µm; (**B**) quantitative results of GT thickness analysis at Days 3 and 7; (*—*p* < 0.05; ****—*p* < 0.0001).

Ischemia/reperfusion applied to animal skin resulted in degree II-III pressure ulcer with significant necrosis of the dermis, subcutaneous fat and muscle at the site of exposure (Figure 4). Data on MSC retention (Figure 3) along with histological assessment of GT (Figure 2) hinted that early stages of its formation may be influenced by CS delivery. We studied histological specimen from Days 3 and 7 using GT thickness as a measure of its growth, relying on previous reports in similar models [23,24,25]. At Day 3, a significant increase of GT thickness (approx. 1.5-fold vs. untreated control) was observed in the “Cell Sheet” group. Significant differences vs. control group were also found in animals treated by MSC suspension, but not MSC secretome. Nevertheless, at Day 7, GT thickness in “Suspension” and “Secretome” groups was significantly higher than in control, while “Cell Sheet” specimen showed the highest GT thickness among all study groups (Figure 4B, Day 7). Later time-points (Days 14 and 21) were characterized by active deposition of connective tissue, GT reduction and were subject to Masson trichrome (Figure 2) described above to evaluate fibrosis.

### 2.5. Vascularization of GT Is Modulated by MSC Secretome and CS

**Figure 5:**
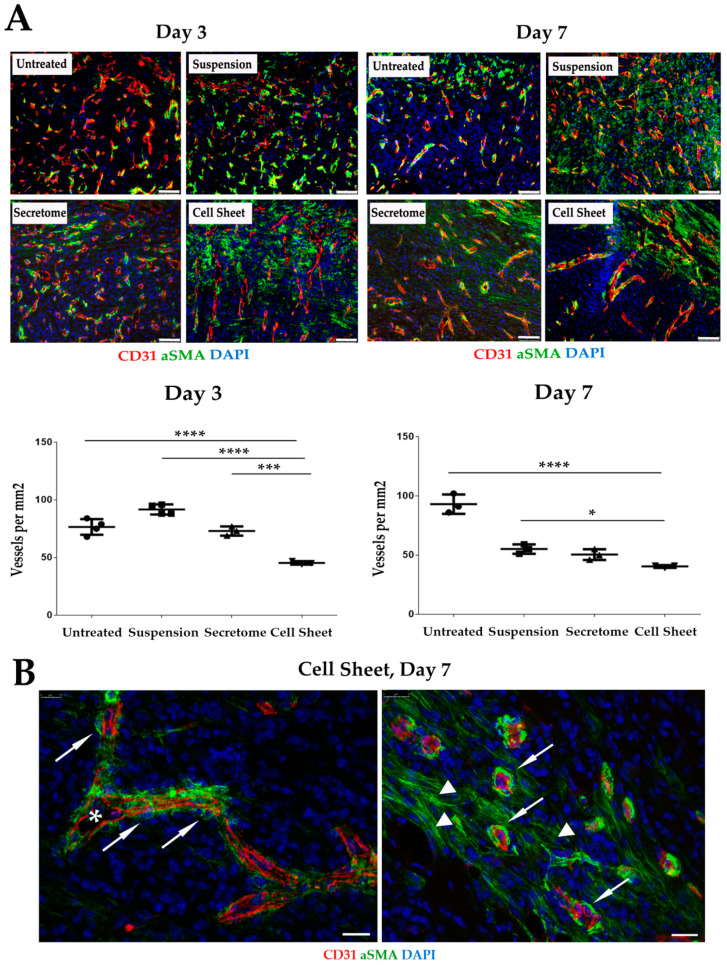
Visualization and quantitative assessment of granulation tissue vascularization in study groups. (**A**) In the “Cell Sheet” group, density of CD31+ blood vessels was reduced (plots at Days 3 and 7). Immunofluorescence, scale bar represents 75 µm; data present results of manual blood vessels counts; (*—*p* < 0.05; ***—*p* < 0.001; ****—*p* < 0.0001); (**B**) representative images from “Cell Sheet” group showing blood vessels in granulation tissue at Day 7 within healing defect (left image) and on the edge of granulation tissue growth (right image). Arrows indicate αSMA-positive mural cells adjacent to CD31+ blood vessels with lumen; triangles mark αSMA-positive myofibroblasts. Immunofluorescence, scale bar represents 75 µm.

Vascularization of GT is a crucial parameter reflecting the degree of its maturation [26]. We used sections from healing ulcers to visualize blood vessels by antibodies vs. CD31 and αSMA (Figure 5). Density of blood vessels in GT at Days 3 and 7 were significantly reduced in the “Cell Sheet” specimen compared to all other groups (Figure 5, plots). Vascularization pattern of GT in “Cell Sheet” and “Secretome” groups showed a high prevalence of relatively large (up to 50 µm in diameter) stabilized vessels with visible lumen and endothelial layer covered by αSMA-positive mural cells (Figure 5B). It should be noted that within the damage site, αSMA-positive cells are represented not only by smooth muscle cells of blood vessels but also by myofibroblasts abundant in GT until scar is formed.

### 2.6. Assembly of MSC in Cell Sheets Increases Secretion of Growth Factors Involved in Blood Vessel Maturation

**Figure 6:**
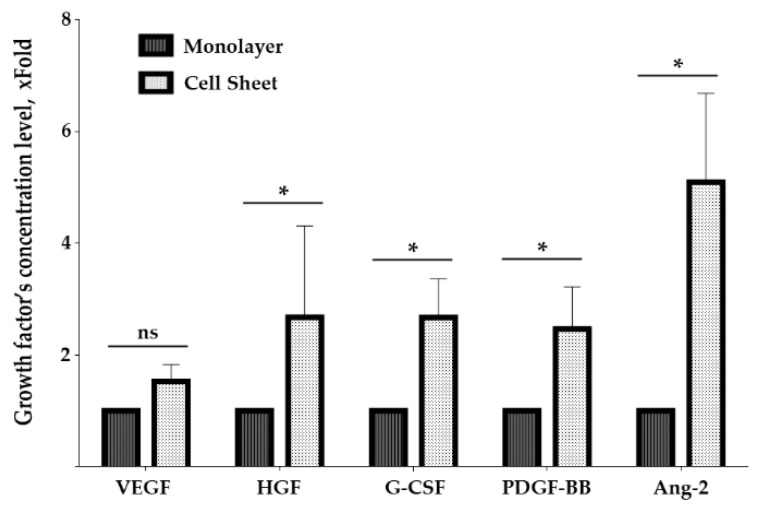
Changes of growth factors production by MSC in cell sheet and monolayer culture. Graph demonstrates assayed protein concentration change expressed as fold increase vs. monolayer culture; data presented as Median (25;75); *—*p* < 0.05, Mann–Whitney.

Short retention of CS along with changes in vascularization of underlying tissue suggested a trigger-like paracrine effect and hinted at evaluation of angiogenesis-related growth factors in CS secretome. At this point, we focused on human cell cultures to evaluate properties of homologous MSC from human adipose tissue, the proposed allogeneic source of cells for further practical development of CS-based treatment for pressure ulcer. Using primary human adipose tissue MSC isolated from three donors and cultured in monolayer or CS, we assayed concentration of growth factors and cytokines in conditioned culture medium. To ensure relevant normalization of results, we took into account the higher density of MSC in sheets compared to confluent monolayer and used PicoGreen DNA-assay for nuclei to normalize change folds. The graph in Figure 6 shows changes of VEGF165, HGF, G-CSF, PDGF-BB and Ang-2 contents in medium samples from CS and monolayer. In CS, increase of angiopoietin-2 was the most profound change (5.1 fold), while increase of a potent sprouting angiogenic factor VEGF165 did not reach statistical significance. It is worth noting that the increase of PDGF-BB reached 2.5 fold—thus, CS secretome was enriched with a crucial factor for pericyte recruitment and vessel maturation.

### 2.7. RNA-Sequencing of Cell Sheets Shows Significant Changes of MSC Transcriptome Profile Compared to Monolayer Culture

For RNA-sequencing experiments, an immortalized line of adipose-derived human MSC (ASCtelo52, ATCC, USA) was used to minimize donor-dependent variability. Assembly of CS was carried out in AdvanceStem medium as previously described [4] and dense monolayer (90% confluent) cultured under the same conditions was used as a control.

**Figure 7:**
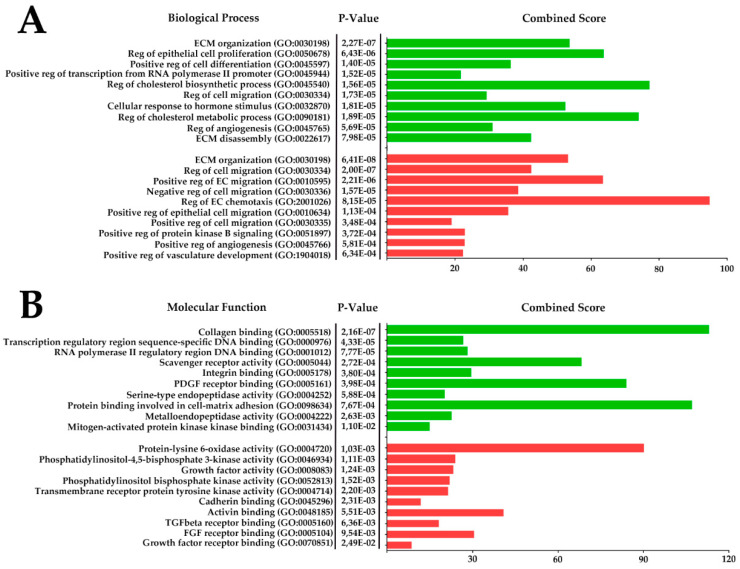
Presentation of RNA-sequencing results mapped using Gene Ontology biological process (**A**) and molecular function (**B**). Data from libraries obtained after RNA-sequencing of monolayer MSC and MSC-based CS were normalized and upregulated (green) and downregulated (red) transcripts were mapped using Gene Ontology. The top 10 *p*-value clusters with a minimum of 3 overlaps after GO mapping were supplemented by combined score calculation (Enrichr). Reg—regulation; ECM—extracellular matrix; EC—endothelial cells; SMC—smooth muscle cell.

RNA-sequencing of MSC monolayer and CS provided libraries that were normalized within each sample as reads per kilobase million (RPKM), and obtained data were used to calculate fold change for each transcript. Using Gene Ontology (GO), we mapped all transcripts with ≥2-fold increment or decrement in CS. The top 10 annotated biological process (BP) and molecular function (MF) GOs with statistically significant *p*-value are presented in Figure 7, completed by a list of genes belonging to each annotated cluster (Supplementary Tables S1 and S2). Notable GOs and results of analysis are overviewed in Discussion and support a general impression of MSC functional shift towards cell-to-ECM interaction, blood vessel maturation and reduction of endothelial cell migration/chemotaxis accompanied by antifibrotic factors (Figure 7).

Additional analysis of trancriptional factor (TF) activity signature was performed using HOCOMOCO database to predict potential transcriptional regulators that may increase their activity due to microenvironment changes during assembly of CS. This provided an array of putative TFs showing increased activity that correlates with their target genes upregulation obtained from our RNA-sequencing data (Table 1).

## 3. Discussion

Animal test demonstrated the efficiency of MSC-based sheets to accelerate pressure ulcer closure. The “Cell Sheets” group was the only one to reach statistical significance vs. untreated control at Day 21 and was clearly superior to suspended MSC (Figure 1) despite CS’s short-lived presence limited by 3–7 days. It should also be noted that at Day 14, CS showed significant improvement vs. MSC secretome, yet by Day 21, this difference failed to reach statistical significance. Another finding to be clarified is the drastic decline of pressure ulcer size between Days 14 and 21 in the CS group. Indeed, at Day 21, ulcer relative size in “Cell Sheet group” was 1:9 of values at Day 14 (Figure 1B, plots). In rodents, a healing skin wound undergoes contraction (typically 10–14 days after damage), which provides a vivid picture for macroscopic assessment. We suggest that during the 3rd week of healing, GT underwent active resorption and strong contraction that was preceded by its accelerated growth (1st week) and maturation (2nd week), with collagen deposition visualized by Masson staining (Figure 2). Effective contraction depends on the presence on myofibroblasts with αSMA-enriched cytoskeleton and requires ECM elasticity that can change due to proteolysis or cross-linking [27]. Further results demonstrated that CS have upregulated a number of enzymes involved in ECM remodeling and turnover, yet accurate mechanism of events observed during the 3rd week of healing is to be investigated in detail.

The most striking finding was that CS delivery induced profound recovery of dermis structure with its appendages, resulting in obvious hair growth at the site of pressure ulcer and histology, confirming the presence of glands within the dermis (Figure 1, Figure 2, Figure S1 and Supplementary Material M1). This was observed exclusively after CS application and three out of four animals of this group had skin microanatomy resembling normal besides residual muscle and fat loss. The latter also indicated that specimens were grafted at the site of pre-existing pressure ulcer, which is characterized by slowly recovering loss of muscular and adipose layers. 

This finding is yet to be clarified using a more accurate method to establish whether hair has re-grown, indicating full-scale regeneration via activation of progenitors or reprogramming. Another option is that dermal layers were drawn towards the center of the defect which has not been occupied by scar mass due to specific CS influence on remodeling of GT. Thus, at the moment, we may claim successful healing induced by CS application, yet the degree of involvement of antifibrotic and regenerative mechanisms, cell proliferation or reprogramming is subject to further investigation. 

Injection of MSC suspension failed to stimulate ulcer closure compared to untreated control group (Figure 1 and Figure 2). Initially, we attributed this outcome to a well-documented low survival of suspended MSC [28]. However, subsequent analysis (Figure 3) revealed that PKH26-labeled MSC injected to lesion remained up to Day 21. In our previous study [12] we found MSC retention at Day 14, and similar data were obtained in skin injury model by Yu et al. [29]. The relevance of this finding should be established in larger animals with skin anatomy resembling human, but lack of MSC survival is unlikely the reason for the poor therapeutic outcome. 

Analysis of Masson stain supported trigger effect of CS application. We found that CS application may accelerate active growth of GT at initial phases of healing (Figure 4), and by Day 7 animals from the “Cell Sheet” group had the maximum thickness of GT while suspended MSC and secretome showed concordant significant increase vs. untreated control. However, if we address the set of data from PKH26-labeled CS suggested we shall find that between Days 3 and 7 the construct was either resorbed (Figure 3), but more likely mechanically removed with detaching scab or during change of wound dressing.

Thus, absence of CS (Figure 3) starting from Day 7 marginally excluded its direct contribution to GT thickness, and enlargement of GT that was induced at the early phase (Day 3) continued even after CS was lost between Days 3 and 7. Furthermore, even at Day 3, when CS was still attached, its presence could not affect morphometry data as far as GT thickness measurement did not include superficial layers of wound bed (see panel markup in Figure 4A). This set of data along with observed efficacy of MSC secretome strengthens the assumption that effects of CS therapy were triggered by this early-time intervention of paracrine nature. Short-lived presence of CS may be a disappointing conclusion in terms of MSC delivery, yet rapid clearance of the construct is beneficial for clinical safety, and lack of cell integration minimizes tumorigenic risks.

Taking into account the data from later time points (Days 14 and 21) where residual GT was marginally absent in CS-treated animals (Figure 2), we suggested that its early increase in thickness after CS application may be followed by resorption or ECM disassembly, resulting in minimum scarring that facilitates more effective healing with skin appendages and hair present in the specimen at Day 21.

Observed changes in GT growth and remodeling depend on its vascularization. Indeed, until GT matures into a scar, it has abundant numbers of blood vessels to facilitate immune cell migration, nutrition and clearance of debris [30]. Thus, we connected signs of scar-free healing in “Cell Sheet” animals with GT vascularization and assessed this parameter in histology sections.

The pattern of GT vascularization in the CS group had an incline towards formation of large vessels with a lumen (up to 50 µm in diameter) and a layer of endothelial cells surrounded by αSMA-positive mural smooth muscle cells (Figure 5B). These large blood vessels represent stabilized arterioles or veins, and shift towards vessel maturation [31] was accompanied by a vivid decline of smaller blood vessel counts in specimen from CS-treated animals at Day 3. “Cell Sheet” group showed significantly lower vascular density than “Secretome” at Day 3, while by Day 7, this difference did not reach significance, indicating a comparable drop of GT vascularization by smaller-size vessels (Figure 5A).

Explanation of how maturation of blood vessels may contribute to less scarring mainly relates to mural cells (pericytes and smooth muscle cells) localization and functional status. When they are attracted to endothelial cells basal membrane formation begins by active production of laminins and collagen IV by both cell types [32]. After deposition of basal membrane mutual influence of the two cell types inhibits endothelial proliferation, induces quiescence of pericytes and normalizes permeability [33]. This results in arrest of plasma proteins leakage and confines the pericytes within mural compartment. According to recent studies pericytes exfoliated from blood vessels are crucial participants of fibrosis [34,35] and formation of a stable vasculature in GT diminishes “roaming” pericytes and vascular leakage. Several studies in skin wounds have shown angiogenic response in normal wounds may exceed what is needed for optimal repair and suppression of vascular growth by angiogenesis inhibitors reduces severity of fibrosis in skin lesions [36].

This explanation was concordant with the analysis of angiogenesis-related factors produced in vitro by MSC and MSC-based CS (Figure 6). Angiogenesis is controlled by a balance of its activators and inhibitors, and each stage is mediated by a specific set of growth factors, proteases and cytokines. At initial stages, the most important and strong angiogenic factor is VEGF165, which increases vascular permeability, endothelial proliferation and branching. At later stages, PDGF-BB is necessary for stabilization by recruitment of pericytes from surrounding stroma. Thus, observed increase of PDGF-BB production by CS may contribute to stabilization of vasculature in GT underlying the transplanted construct. At the same time, production of VEGF165 in CS did not increase compared to monolayer MSC, which also favors more stable vasculature and reduction of increased permeability associated with effects of VEGF (Figure 6).

Other important participants are angiopoietins that activate, maintain and harness angiogenic response when blood vessel is formed. We found angiopoietin-2 (Ang-2) strongly increased in CS secretome (5.1-fold vs. monolayer), and typically Ang-2 is presented as a sprouting factor. Ang-2 acts via Tie-2 receptor to destabilize pericyte–endothelium interaction, increasing the endothelial cells’ sensitivity to inflammatory and angiogenic factors. However, the peculiar ability of Ang-2 to block sprouting and induce apoptosis of endothelial cells is described under the low production of VEGF165 [37,38]. Basically, when capillary sprouting is induced by factors besides VEGF165, Ang-2 may contribute to the arrest of angiogenesis rather than support it [39]. Furthermore, Ang-2 may also bind Tie-2 expressed on pericytes, and recent elegant work by Teichert et al. [40] has shown that depletion of Tie-2 resulted in a pro-angiogenic effect. This work suggested that activation of Tie-2 in pericytes is crucial for reciprocal control of vascular stability independent of ligand type—Ang-1 or Ang-2.

Overall, this profile of angiogenesis regulators produced by CS suggested a shift of its biological potency towards “stabilizing” rather than “stimulating” action on endothelial cells. Supporting our hypothesis, we also found HGF and G-CSF significantly upregulated in CS secretome (approx. 2.6-fold for both factors). These factors contribute to blood vessel maturation and leakage stop [41] and may indicate a switch of secretome modality to blood vessel stabilization.

Our conclusion regarding the shift in MSC biological potency induced by CS formation was supported by analysis of RNA-sequencing data (Figure 7; Supplementary Tables S1 and S2). Results of GO analysis showed that in CS, upregulated genes were annotated to clusters with high *p*-value that contain proteins important for cell–ECM interactions (GO:000518 “Collagen binding” and GO:0098634 “Protein binding involved in cell-to-matrix adhesion”). A high number of secreted proteolytic enzymes and their inhibitors were annotated to BP clusters related to ECM maturation, turnover and change of its composition in normal during healing after injury (GO:0030198 “ECM organization” and GO:0022617 “ECM disassembly”). Secreted proteases (including MMPs 2, 11 and 16) and upregulated CXCL-motif activators of cell migration (GO:0030334 “Reg of cell migration”) may potentially contribute to observed rapid thickening and remodeling of GT in early stages after CS transplantation. Some of GO:0030198 “ECM organization” have also been downregulated with a high combined score for this cluster. This is a typical consequence of GO annotation redundancy when the same GO may be significantly up- and downregulated during analysis with different transcripts falling into one of these categories due to high fold increment or drop. However, the majority of downregulated transcripts were related to MSC integrins and ECM proteins (COL4, ICAMs, THBS1) that comprise CS structure rather than those that can be secreted and influence underlying GT during healing.

Among upregulated transcripts, peculiar clusters were annotated as GO:0045540 “Reg of cholesterol biosynthesis process” and GO:0090181 “Reg of cholesterol metabolic process” rich with enzymes controlling cholesterol biosynthesis and alcohol biogenesis. More detailed analysis showed the majority of them were responsible for fatty acid metabolism known as negative regulators of angiogenic response in normal tissue and tumors including suppression of VEGF-induced capillary sprouting.

Analysis of downregulated genes mapped multiple proteins known to drive angiogenesis and active endothelial proliferation. Among all downregulated BPs, GO:2001046 “Reg of EC chemotaxis” containing NOTCH1 and FGFs 1 and 2 showed the highest combined score (Figure 7). Another vivid illustration of this is BP analysis of downregulated transcripts showing decline of factors that stimulate endothelium sprouting, including WNT5A [42] and SERPINE1. Finally, MF mapping of downregulated transcripts showed enrichment of a cluster annotated as GO:0004720 “Protein-lysine 6-oxidase activity”, containing LOX-family proteins mostly known to be involved in ECM organization and gaining attention as a therapeutic target. Recent data show that LOX-encoded protein-lysine 6-oxidase is a positive regulator of both-angiogenesis and tissue fibrosis [43].

HOCOMOCO dataset (Table 1) showed significant activation of a TFs repertoire identified indirectly by upregulated targets. Increased activity of specificity protein-1 (SP-1) can be attributed to many factors as far as SP-1 (despite its name) is known for non-specific induction under different stimuli ranging from the mechanical influence and cell cycle to effects of mitogenic growth factors. However, marked activity of inflammation-associated nuclear factor kappa-B1 (NFKB1) was a surprising marker after evaluation of MSC sheets. Concordant increased activity of its heterodimer partner RELA was expected and supported the quality of the performed predictive analysis. At the moment, we lack sufficient evidence to claim a consequence of TFs activation shift, and the mechanism of these changes remains enigmatic prior to additional study. We provide this pool of data to illustrate the magnitude of transcriptional changes after self-assembly of MSC to CS. Nevertheless, transcriptional changes and modifications of differentiation potency of stromal cells within spheroids and other 3D cultures [17,19,20,44] have been reported, giving us ground for further investigation after providing the first piece of evidence that similar events take place in CS.

A potential limitation of our in vitro data obtained in human MSC is that its extrapolation to animal model might be challenging, and validation of proposed mechanisms would require a more detailed study based on presented screening of changes induced by CS assembly. However, our study intended to lay ground for pre-clinical development of a cell-based product for pressure ulcer patients, so we performed tests in human MSC from adipose tissue, which is the most feasible source of allogeneic MSC. This shift creates a certain limitation, yet we believe that we also provide a physiological rationale for a novel product that can be applied in cell therapy.

In vitro tests also should be evaluated keeping in mind that alterations in the repertoire of MSC secretome and transcriptome that may occur after transplantation to damaged tissue are hard to study and reproduce ex vivio. Nevetheless, this point must be taken into account while developing further hypotheses on mechanisms of impact rendered by MSC delivered via injection or CS application. 

## 4. Materials and Methods

### 4.1. Cell Cultures

Human adipose-derived MSC immortalized cell line (ASC52telo) was purchased from ATCC (Manassas, VA, USA) and cultured using AdvanceStem culture medium (Hyclone, USA). Mouse adipose-derived MSC were obtained from subcutaneous adipose tissue of male C57Bl/6 mice as previously described [12]. Briefly, adipose tissue samples were mechanically minced using surgical scissors and subjected to enzymatic digestion using a mixture of type I collagenase (200 u/mL, Worthington, Columbus, OH, USA) and dispase (30 u/mL, Corning, NY, USA) for 60 min at 37 °C. Equal volume of DMEM supplemented by 10% FBS (HyClone, Logan, UT, USA) was added to digested tissue mixture to inactivate the enzymes followed by centrifugation (200× *g*; 10 min). After supernatant was aspirated, the pellet was resuspended in complete growth medium at 5 × 10^3^ cells/cm^2^ and seeded in Petri dishes. Cells were cultured at 37 °C and 5% CO_2_. Upon reaching 80–90% of monolayer density, cells were re-seeded at a 1:4 ratio using 0.05% Trypsin-EDTA (Gibco, Waltham, MA, USA). Mouse MSC were used for in vivo experiment using a syngeneic model to avoid immune rejection while human MSC were used for in vitro studies (RNA-Seq, BioPlex assay) as described below.

### 4.2. MSC Secretome Preparation

To obtain secretome samples, human MSC were cultured in a 100 mm dish in complete growth medium to 80% monolayer. Upon reaching the designated density, cells were washed by Hanks’ solution (3 times, 5 min), and 10 mL of serum-free DMEM was added. Cells were cultured for 7 days in a CO_2_ incubator as described in Section 4.1. At Day 7, culture medium containing secreted fractions was collected, centrifuged at 1500 *g* for 10 min to remove cell debris and used for BioPlex assay or animal tests without freezing.

### 4.3. Fabrication of Cell Sheets from MSC

Mouse primary adipose-derived MSC isolated as described above were used at passage 2–3 for animal test in pressure ulcer model. Animal manipulations and euthanasia procedures were performed in compliance with National and European Union regulations and were approved by the Institutional (Ethics Board for Animal Care) Animal Care and Use Committee (Lomonosov Moscow State University; permit #67; 15 March 2018). For RNA-sequencing and growth factor BioPlex assay, human immortalized line ASC52telo (ATCC, USA) was used at passage 4–5. For both purposes, MSC were seeded in a 12-well uncoated culture plate at 8 × 10^4^ cells/cm^2^ and cultured for 7 days in complete growth medium supplemented by ascorbic acid (50 μg/mL) to promote ECM deposition and assembly. Before the detachment growth medium was removed and CS was washed with warm PBS. After that, CS edges were carefully detached from the plastic using a micropipette tip used as a scrapper, and then whole-mount CS was moved for further transplantation to mice. Preparations for BioPlex assay and RNA-sequencing of human MSC cultures are described in corresponding sections below.

### 4.4. BioPlex Assay of Growth Factors in MSC Monolayer and Cell Sheet Secretomes

MSC-derived secretome samples were collected from cultures at the state of confluent monolayer or CS and were used for multiplex ELISA. For normalization to DNA concentration, cells that remained in the plate after medium collection were lysed by TRIzol reagent (Thermo Fisher Scientific, Waltham, MA, USA). Concentrations of Angiopoietin-2, G-CSF, HGF, PDGF-BB and VEGF were simultaneously evaluated with a multiplex bead-based sandwich immunoassay kit (Human Angiogenesis assay, Bio-Rad Laboratories, Hercules, CA, USA) using Bio-Plex^®^ 200 system (Bio-Rad, USA). Assay was performed following the manufacturer’s instructions. Briefly, 9 distinct sets of fluorescent-dye-conjugated beads with capture monoclonal antibodies specific for each factor assayed were used. Samples or standards were incubated with pre-mixed bead sets in pre-wet 96-well microplate. After incubation and washing, fluorescent detection antibody mixture was added, and after incubation, the samples were washed and resuspended in assay buffer. All values were normalized with respect to DNA concentration and measured with Quant-iT PicoGreen dsDNA Assay Kit (Thermo Fisher Scientific, USA) according to manufacturer’s manual. DNA concentration was a directly proportional function of the number of cells within study limits (calibration was carried out in-house).

### 4.5. RNA Isolation and Transcriptome RNA-Sequencing Analysis

Immortalized human MSC (ASC52telo) were cultured in AdvanceStem medium to obtain monolayer (see Section 4.1) or CS (described in Section 4.3). After completion of culture, they were lysed by TRIzol reagent (Thermo Fisher Scientific, USA) and total RNA was extracted. Quality of RNA was checked using BioAnalyser and RNA 6000 Nano Kit (Agilent, Santa Clara, CA, USA). PolyA RNA was purified with Dynabeads^®^ mRNA Purification Kit (Ambion, Austin, TX, USA), and Illumina library was made from polyA RNA with NEBNext^®^ Ultra™ II RNALibrary Prep (NEB, Ipswich, MA, USA) according to manufacturer’s protocol. Concentrations of nucleic acids in obtained libraries were analyzed by Qubit dsDNA HS Assay Kit (Thermo Fisher Scientific, USA) using Qbit 2.0 equipment. RNA-sequencing was performed on HiSeq1500 at 50 b.p. read length according to manufacturer’s protocols.

Quality control was carried out using the FastQC program, and while analyzing, the source data of the adapters were not found; therefore, programs for their removal were not used. Mapping was performed using bowtie2–2.4.0; to assess changes in the level of gene expression samples were normalized as reads per kilobase million (RPKM). GO gene clustering and annotation of up- and downregulated transcripts was performed using the David 6.8 resource and Enrichr web-interface (https://amp.pharm.mssm.edu/Enrichr/ accessed on 1 July 2020).

For mapping of putative transcription factors that overlap with transcripts obtained after RNA-seq, we used resources of online database HOCOMOCO (https://hocomoco11.autosome.ru accessed on 3 July 2020). To establish the potential for interaction of mapped transcription factors with the putative genes, bash scripts were used to obtain a unique list.

### 4.6. Pressure Ulcer Model

Animal manipulations and euthanasia procedures were performed in compliance with National and European Union regulations and were approved by the Institutional (Ethics Board for Animal Care) Animal Care and Use Committee (Lomonosov Moscow State University; permit #67; 15 March 2018).

For in vivo studies, we used male C57Bl/6 mice 12–14 weeks of age. The pressure ulcer defect was formed according to the protocol described by Stadler et al. [45] with modifications. Briefly, animals were narcotized with isoflurane, hair on the back was shaved and skin was gently pulled to form a fold that was placed between two round magnets (12 mm diameter, Magnetic Source, Castle Rock, CO, USA). Magnets were removed at 12 h, and no pressure was applied for 12 h to form a cycle of ischemia/reperfusion of 24 h (12 h with magnet + 12 h without magnet). Full-thickness skin loss with damage and necrosis of subcutaneous tissue and muscle layer (ulcer stage 3), requiring more than 14 days to heal without outside intervention, required 3 cycles of ischemia/reperfusion. As a result, 2 symmetrical pressure ulcers were obtained in one animal on sides of skin fold that was placed between magnets.

### 4.7. Delivery of MSC Suspension, Secretome or MSC Sheets

At the end of 3 cycles of ischemia/reperfusion, test material was transplanted to animals (day 0). The animals were divided into the following groups (*n* = 12–16 animals per group):

(1) “Suspension”—injection suspended MSC to the edges and bottom of skin defect.
(2) “Secretome”—injection of MSC secretome into the edges and bottom of skin defect.
(3) “Cell Sheet”—application of CS to ulcer surface.
(4) “Untreated”—animals without therapy.

The MSC suspension was administered in the amount of 4–5 injections into the edges and bottom of the wound (10^6^ cells in 150 μL of PBS per defect). Secretome injections were performed in a similar volume (150 μL, 4–5 injections). CS was transplanted to the defect in a drop of PBS, straightened with tweezers and allowed to adhere to lesion surface.

Preliminary assessments using PicoGreen assay have shown that using 100 mm dishes for MSC culture prior to injection and scaling down CS size to 12-well format resulted in nearly dose-equivalent delivery of MSC with CS containing (1 ± 0.12) × 10^6^ cell/construct.

To preserve the cellular material (primarily the CS on the defect surface), transplantation sites in all groups were covered for 3 days with Tegaderm film (3M, St. Paul, MN, USA)-breathable, sterile, transparent and waterproof stickers for closing wounds. All animals were housed in individual cages for 21 days until the end of experiment

### 4.8. Wound Assessment and Histological Examination

Skin defect area was measured at days 3, 7, 14 and 21 in photographs of wound surface at each time-point. Area was calculated using ImageJ software (NIH, Bethesda, MD, USA).

After animal euthanasia, whole-mount specimen of defect (or healed skin in CS group) were isolated and bisected for histological assessment as follows; one half was fixed in formalin, paraffin-embedded, sectioned at 5 µm and stained with hematoxylin/eosin. The other half was placed in Tissue-Tek O.C.T. Compound and frozen in liquid nitrogen and stored at −70 °C, and frozen sections at 6–7 µm were obtained for immunofluorescent staining.

### 4.9. Histological Evaluaton of Healing Processes: Granulation Tissue Formation and Maturation

Sections stained by hematoxylin-eosin/Masson’s Trichrome were used for morphometry to assess thickness of granulation tissue on days 3 and 7 (days 14 and 21 showed remodeling of granulation tissue and were not included in data analysis). Since the composition of the defect was qualitatively different in the groups on Days 14 and 21, collagen deposition was evaluated by calculating its percentage of dyed collagen fibers (blue) to the total defect area using an ImageJ-associated color deconvolution plugin [46]. Additionally, the percentage of immature GT zones was evaluated by calculating the ratio of the measured areas to the defect area. Margins of the defect corresponded to edges of ulcer characterized by loss of subcutaneous muscle. ImageJ software (NIH, USA) was used for described morphometry.

### 4.10. Immunofluorescent Staining and Vessel Density Analysis

Frozen tissue sections (at days 14 and 21) were fixed in 4% formaldehyde, blocked by 10% normal goat serum, washed and incubated with rat-anti-mouse CD31 (Biolegend, Cat#102401) and rabbit-anti-αSMA (Abcam, Cat#ab32575) antibodies. Then, slides were washed in PBS and incubated in a mixture of goat anti-mouse AlexaFluor 594 and goat anti-rabbit AlexaFluor 488 conjugated antibodies. At the end of incubation, slides were washed, counterstained with DAPI and mounted.

Microphotographs of sections were taken under ×100/×200 magnification in 3 random fields of view (FOV) per section (DMi8, Leica, Germany). Vessel counts included CD31-positive structures per FOV; αSMA staining was used to detect large vessels and myofibroblasts in granulation tissue. Structure counts were obtained using ImageJ software (NIH, USA). Classification of blood vessels from Müller et al. with adaptations was used for analysis of blood vessel type [31]. 

### 4.11. PKH26 Labelling and Subsequent Detection of Transplanted Cells in Histological Preparations

Cells were labeled by a commercial PKH26 dye (Cat#PKH26GL, Sigma, USA), according to manufacturer’s protocol. The cell suspension was labeled immediately before the transplantation; for the CS group, cells were labeled before assembly of CS. To detect a fluorescent signal, tissue samples were frozen in Tissue-Tek O.C.T. Compound medium and sectioned. Immediately after microscopy, slides were dried at RT, and we visualized PKH26-positive cells using a fluorescence microscope (Leica DMi8, Leica Microsystems GmbH, Mannheim, Germany). For a more detailed study of cells’ localization, sections were fixed and were immunolabeled to detect blood vessels and myofibroblasts followed by nuclear stain by DAPI.

### 4.12. Statistical Analysis

Data are expressed as mean ± standard deviation. To compare >2 groups, we used analysis of variance (ANOVA) and Tukey criterion for multiple comparisons, or Kruskal–Wallis criterion supplemented by Dunn criterion (as a nonparametric ANOVA). Differences were considered significant at *p* < 0.05. Analysis was performed using Prism 7.03 (GraphPad Software).

## 5. Conclusions

Pressure ulcer healing is effectively induced by MSC secretome or CS application, while suspended cells performed poorly despite its long-lasting retention in the tissue. The most peculiar finding is that CS was the only group that showed minimal scarring with growth of hair and dermal glands in histological sections of pressure ulcer site at Day 21. The nature of this phenomenon should be established including the mechanism of defect healing via activation of regenerative capacity or healthy skin elements being drawn to the site of resorbed ECM.

We propose that CS acts via a trigger mechanism mediated by its paracrine factors released at early stages of healing to induce recovery of skin structure after pressure ulcer. Effects of CS involve stabilization of vasculature and rapid GT growth followed by a specific pattern of remodeling that results in ECM resorption, but not scarring. This anti-fibrotic influence facilitates full closure of defect and observed healing of dermis.

The spectrum of growth factors and cytokines produced by MSC after CS assembly can be described as a pattern for activation of blood vessel stabilization (PDGF-BB, HGF, G-SCF, Ang-2) rather that sprouting (VEGF165), which was concordant with vascular patterns in corresponding study groups.

The proposed mechanism relies on changes of secretome, yet transcriptomic profile and TFs activity hint at future evaluation of potential MSC reprogramming within CS. RNA-sequencing profiles also show that transcriptome of MSC in CS is enriched for biological processes and molecular function that support the expression of factors stabilizing blood vessels and limiting endothelial sprouting. Future studies will address our findings regarding the potential role of ECM remodeling induced by delivered CS that may explain changes of GT maturation and its quick resorption resulting in the recovery of skin structure with its appendages (hair and glands).

A conceptual study limitation is a lack of understanding of immune cells interaction with CS and how it may modulate the response of immunocompetent cells as far as they present important generators of angiogenic stimuli in damaged tissue [47,48]. Successful tissue repair driven by MSC is impossible without immune response, and this point is sto be addressed in future studies.

The findings are potentially of importance for both fundamental understanding of MSC participation in regeneration as well as for development in tissue engineering methods addressing an unmet medical need—pressure ulcer.

## Figures and Tables

**Table 1:** Transcription factors with significantly increased activity evaluated by expression of their targets in MSC-based cell sheets compared to monolayer MSC. TF—transcription factor; entries ranked by Q-value and *p*-value; TFs with 4 and more overlapping targets presented.

Rank	TF	Overlapped Genes N	List of Overlapped Genes
1	SP1	28	C4A, AGTR1, EDNRB, AGT, PPL, MMP2, TNC, EGR1, LSP1, CCND2, TNFSF10, MME, FBLN1, CYP27A1, SOX9, COMP, SLC39A8, COL18A1, C4B, SREBF1, HGF, TCN2, ISG20, APOE, SOD2, CYP19A1, PTN, CHI3L1
2	NFKB1	22	EGR1, BGN, CYP19A1, PTGFR, TNC, SOD2, ADORA1, CXCL12, TNFSF13B, MMP2, CD74, FGF7, IRF4, AGT, SLC25A27, PLA2G2A, CCND2, BCL2L11, IRF7, VCAM1, A2M, TNFSF10
3	RELA	20	IRF7, EGR1, FGF7, ADORA1, CXCL12, MMP2, SLC25A27, SOD2, VCAM1, BCL2L11, TNFSF10, PLA2G2A, TNC, CD74, CYP19A1, CCND2, PTGFR, AGT, BGN, IRF4
4	FOXO3	8	BCL6, TXNIP, TNFSF10, BCL2L11, CCND2, VEGFA, CDKN2B, VEGFB
5	USF1	8	FMO3, TCN2, AGT, CYP19A1, SLC1A3,LIPC, CTSD, ISG20
6	SREBF1	5	LRP1, ACACB, LDLR, FASN, CIC
7	STAT3	10	CCND2, CFB, PROS1, HGF, MMP2, CYP19A1, A2M, CHI3L1, DIRAS3, BCL6
8	HIF1A	12	TGFB3, TLR6, VEGFA, ARNT, TIMP2, SOCS1, VEGFB, MMP2, CXCL12, EDNRB, ACE, AGTR1
9	FOXO1	4	TNFSF10, EGR1, ANGPT2, TXNIP
10	JUN	10	DCN, VCAM1, CYP19A1, MMP2, SOD2, PTN, TNC, MGP, FGF7, LBP
11	DNMT1	4	ESR1, IL32, CDKN2B, VEGFA
12	SP3	8	FBLN1, HGF, SLC1A3, ACE, TCN2, MMP2, AGTR1, CYP27A1
13	CREB1	7	NR4A3, AQP3, BCL2L11, BDKRB2, SOX9, MMP2, CYP19A1
14	MYC	7	VEGFA, JUNB, HLA-B, TFAP4, SHMT1, MST1, BCL2
15	ESR1	6	ESR1, BCL2, VEGFA, ZEB1, CEBPB, JUNB
16	ETS2	4	EGR1, ANGPT2, MMP2, TNC
17	WT1	5	WTAP, BCL2, VEGFA, VEGFB, JUNB
18	ATF4	4	IRF7, HRK, DDIT4, APOE
19	PPARA	4	TXNIP, SOD2, G0S2, CD36
20	SPI1	5	JCHAIN, BCL6, MME, CTSS, CTSK
21	MYCN	4	EFNB3, CTSD, MXI1, CLU
22	USF2	4	TCN2, CTSD, CYP19A1, LIPC
23	HDAC1	5	HLA-DRA, EGR1, CCND2, TXNIP, SFRP1
24	STAT1	4	UPP1, XAF1, STAT2, MUC1
25	BRCA1	4	ESR1, IRF9, VEGFA, DDIT3
26	TP53	8	BDKRB2, GPNMB, PDGFRB, AQP3, EGR1, PMAIP1, CTSD, MMP2
27	ETS1	5	DUSP6, ANGPT2, EGR1, TMEM158, TNC
28	YY1	5	SAP30, VWF, LSS, VEGFB, LDLR
29	BRCA1	4	EGR1, CTSD, IRF7, CYP19A1

## Supplementary Materials

Supplementary materials can be found at https://www.mdpi.com/1422-0067/21/15/5567/s1.

## Abbreviations

Ang-1	Angiopoietin-1
Ang-2	Angiopoietin-2
ANOVA	Analysis of Variation
αSMA	Alpha smooth muscle actin
BP	Biological process
CS	Cell sheet
DEG	Differentially expressed genes
DNA	Deoxyribonucleic acid
ECM	Extracellular matrix
ELISA	Enzyme-linked immunosorbent assay
FBS	Fetal bovine serum
FGF	Fibroblast growth factor
GAPDH	Glyceraldehyde 3-phosphate dehydrogenase
G-CSG	Granulocyte-colony stimulating factor
GO	Gene ontology
GT	Granulation tissue
HGF	Hepatocyte growth factor
LOX	Lysyl oxidase
MF	Molecular function
MSC	Mesenchymal stromal cell
NFKB1	Nuclear factor κB
PDGF	Platelet-derived growth factor
RELA	REL-associated protein
RNA	Ribonucleic acid
SP-1	Specificity protein 1
TF	Transcription factor
TGF	Transforming growth factor
Tie-2	Endothelial specific receptor tyrosine kinase
VEGF	Vascular endothelial growth factor
