# Supplementary material for: Cell Sheets from Adipose Tissue MSC Induce Healing of Pressure Ulcer and Prevent Fibrosis via Trigger Effects on Granulation Tissue Growth and Vascularization
Source: Int J Mol Sci. 2020 Aug 4;21(15):5567. doi: 10.3390/ijms21155567 (PMC7432086; doi:10.3390/ijms21155567)
Supplement: Supplementary file 1 [file ijms-21-05567-s001.zip › supplementary/Supplementary R2/Supplementary Table S1.pdf]

Supplementary Table A1

RNA Sequencing/Biological Process

Upregulated

	Term	P-value	Combined Score	Overlap	Genes
1	extracellular matrix organization (GO:0030198)	2,27E-07	53,264	23/229	CSGALNACT1;COL18A1;COL14A1;LUM;MMP2;LAMA4;BGN;TNC;HTRA1;LAMB1;NID1; CTSS;DCN;MMP11;ADAMTS5;MMP16;CTSK;ITGA10;COL6A2;ITGA11;MFAP2;TIMP2;COL6A3
2	regulation of epithelial cell proliferation (GO:0050678)	6,43E-06	63,309	11/72	OSR2;FGF7;SFRP1;CDKN2B;NR4A3;DAB2IP;LAMB1;SOX9;MTSS1;ZFP36L1;VEGFA
3	positive regulation of cell differentiation (GO:0045597)	1,40E-05	35,953	18/194	WWTR1;IFITM1;CEBPB;JUND;PKDCC;HGF;AXIN2;MEDAG;ZFP36L1; SFRP1;BOC;TMEM119;SOX9;CD36;JUNB;ZNF385A;CDON;CMKLR1
4	positive regulation of transcription from RNA polymerase II promoter (GO:0045944)	1,52E-05	21,307	47/848	CEBPB;INSIG1;GLIS3;FOXO4;LDB2;MEOX2;GLI3;DBP;IFI16;PRDM16;NAMPT;SOX9;JUNB; KDM6B;WWOX;EGR1;WWTR1;IL33;OSR2;SREBF1;CDKN2B;JUND;HGF;DAB2IP;NR1H3;ESR1; PBX1;SREBF2;DCN;NFATC4;RUNX1;RGMA;VEGFA;KAT2B;PLSCR1;ZEB2;NR4A3;IRF4; HAND2;DDIT3;CREBRF;IRF7;F2RL1;MAML3;LPIN1;OGT;ATF4
5	regulation of cholesterol biosynthetic process (GO:0045540)	1,56E-05	76,747	8/40	SREBF1;SCD;FASN;MVD;APOE;ACACB;LSS;SREBF2
6	regulation of cell migration (GO:0030334)	1,73E-05	28,870	24/316	PDGFRB;PDGFRA;IFITM1;ACE;MCTP1;HGF;STC1;SEMA3E;LAMB1;ADARB1;LDB2;PODN; SOD2;VEGFA;AMOT;IGSF10;SFRP1;FRMD5;CXCL12;GPNMB;ENPP2;F2RL1;ROR2;SGK1
7	cellular response to hormone stimulus (GO:0032870)	1,81E-05	52,044	11/80	ABCC2;JUND;NR4A3;AKR1C1;KLF9;AKR1C3;AKR1C2;SLIT3;JUNB;CTSS;ZFP36L1
8	regulation of cholesterol metabolic process (GO:0090181)	1,89E-05	73,578	8/41	SREBF1;SCD;FASN;MVD;APOE;ACACB;LSS;SREBF2
9	regulation of angiogenesis (GO:0045765)	5,69E-05	30,628	16/177	NPR1;SERPINF1;VEGFB;DAB2IP;SEMA3E;VASH1;FOXO4; THBS2;DCN;AMOT;RUNX1;VEGFA;SFRP1;GPNMB;ADGRA2;CHI3L1
10	extracellular matrix disassembly (GO:0022617)	7,98E-05	41,935	10/78	ADAMTS5;MMP11;MMP16;CTSK;MMP2;TIMP2;HTRA1;NID1;CTSS;DCN

Downregulated

	Term	P-value	Combined Score	Overlap	Genes
1	extracellular matrix organization (GO:0030198)	6,41E-08	52,809	28/229	DDR1;SPARC;ELN;SERPINE1;ICAM2;LOXL3;PDGFA;LAMC2;FGF2;THBS1;HAPLN1;LOXL2; SCUBE3;HAS3;MMP1;ITGA3;ITGA2;MMP3;ADAM19;COL4A2;LOX;MMP15;COL4A1;COL5A3; COL8A1;ITGA6;TGFBI;ITGA5
2	regulation of cell migration (GO:0030334)	2,00E-07	42,005	33/316	LRRC15;CLIC4;SEMA7A;NOTCH1;CITED2;SERPINE1;SEMA3B;PDGFA;PIK3CD;PTPRJ;PRKX; LAMC2;SEMA3F;FGF1;THBS1;MYPN;PODXL;WNT5B;HSPA5;TPM1;ACTN4;RHOC;GTPBP4; RHOB;TIAM1;SNAI1;MYADM;PLXNB3;PLXNB2;AMOTL2;ITGA6;SINHCAF;ENG
3	positive regulation of endothelial cell migration (GO:0010595)	2,21E-06	63,070	13/70	MAP2K3;FOXC2;SPARC;WNT5A;FGF1;FGF2;THBS1;HIF1A;RHOB;ATOH8;TEK;CALR;MET
4	negative regulation of cell migration (GO:0030336)	1,57E-05	38,152	16/121	NDRG4;CLIC4;CITED2;SERPINE1;TPM1;PTPRJ;FGF2;GTPBP4;RHOB;PBLD;PLXNB3; CALR;SINHCAF;TRIB1;PFN2;ENG
5	regulation of endothelial cell chemotaxis (GO:2001026)	8,15E-05	94,423	5/13	NOTCH1;FGF1;THBS1;FGF2;MET
6	positive regulation of epithelial cell migration (GO:0010634)	1,13E-04	35,217	11/74	DOCK5;FOXC2;SPARC;WNT5A;ATOH8;TEK;CALR;FGF1;THBS1;HIF1A;RHOB
7	positive regulation of cell migration (GO:0030335)	3,48E-04	18,790	20/221	LRRC15;DOCK5;SEMA7A;NOTCH1;WNT5B;HSPA5;SEMA3B;PDGFA;PIK3CD;LAMC2; ACTN4;SEMA3F;FGF1;HIF1A;THBS1;TIAM1;PODXL;SNAI1;MYADM;ITGA6
8	positive regulation of protein kinase B signaling (GO:0051897)	3,72E-04	22,698	14/127	PDGFA;PIK3CD;PTPRJ;TXN;FGF1;FGF2;THBS1;FGF5;KITLG;ERBB3;KIT;TEK;MET;ENG
9	positive regulation of angiogenesis (GO:0045766)	5,81E-04	22,637	12/103	FOXC2;SERPINE1;WNT5A;ITGA5;ANGPTL4;TEK;FGF1;FGF2;HIF1A;THBS1;PGF;RHOB
10	positive regulation of vasculature development (GO:1904018)	6,34E-04	22,155	12/104	SERPINE1;WNT5A;KIT;ANGPTL4;TEK;FGF1;FGF2;HIF1A;THBS1;PGF;RHOB;ENG
