# Supplementary material for: Cell Sheets from Adipose Tissue MSC Induce Healing of Pressure Ulcer and Prevent Fibrosis via Trigger Effects on Granulation Tissue Growth and Vascularization
Source: Int J Mol Sci. 2020 Aug 4;21(15):5567. doi: 10.3390/ijms21155567 (PMC7432086; doi:10.3390/ijms21155567)
Supplement: Supplementary file 1 [file ijms-21-05567-s001.zip › supplementary/Supplementary R2/Supplementary Table S2.pdf]

Supplementary Table A2

RNA Sequencing/Molecular Function

Upregulated

	Term	P-value	Combined Score	Overlap	Genes
1	collagen binding (GO:0005518)	2,16E-07	112,523	11/52	MRC2;ECM2;COL14A1;LUM;CTSK;ITGA10;ITGA11;PCOLCE;NID1;PODN;CTSS
2	transcription regulatory region sequence-specific	4,33E-05	26,237	22/292	EGR1;SREBF1;ZNF395;CEBPB;NR1H3;AEBP1;ESR1;PBX1;GLI3;RUNX1;PER3;NR4A3;DBP; NFIL3;DDIT3;TRPS1;HAND2;MXI1;CREBRF;SOX9;ZNF589;ATF4
3	RNA polymerase II regulatory region DNA binding (GO:0001012)	7,77E-05	27,742	17/201	EGR1;SREBF1;CEBPB;NR1H3;AEBP1;PBX1;GLI3;RUNX1;NR4A3;DBP;NFIL3;TRPS1; HAND2;MXI1;CREBRF;ZNF589;ATF4
4	scavenger receptor activity (GO:0005044)	2,72E-04	67,743	5/21	COLEC12;LRP1;ACKR3;SSC5D;CD36
5	integrin binding (GO:0005178)	3,80E-04	29,041	10/94	ADAMTS5;FRMD5;ECM2;CXCL12;GPNMB;FAP;LTBP4;EMILIN1;S1PR3;CD177
6	platelet-derived growth factor receptor binding (GO:0005161)	3,98E-04	83,491	4/13	PDGFRB;PDGFRA;IL1R1;VEGFA
7	serine-type endopeptidase activity (GO:0004252)	5,88E-04	19,735	15/196	CFD;C1S;C1R;MMP2;HTRA1;CTSS;C4B;C3;DPP4;C4A;MMP11;FAP;CTSK;CTSD;CFB
8	protein binding involved in cell-matrix adhesion (GO:0098634)	7,67E-04	106,564	3/7	ITGA10;ITGA11;EMILIN1
9	metalloendopeptidase activity (GO:0004222)	2,63E-03	22,181	7/65	ADAMTS5;MMP11;MME;MMP16;FAP;MMP2;ADAM33
10	mitogen-activated protein kinase kinase binding (GO:0031434)	1,10E-02	14,427	6/65	PDGFRB;PDGFRA;EPHA4;ACE;DAB2IP;ROR2

Downregulated

	Term	P-value	Combined Score	Overlap	Genes
1	protein-lysine 6-oxidase activity (GO:0004720)	1,03E-03	89,675	3/6	LOX;LOXL3;LOXL2
2	phosphatidylinositol-4,5-bisphosphate 3-kinase activity (GO:0046934)	1,11E-03	23,471	9/68	FGF5;KITLG;ERBB3;KIT;PIK3CD;PDGFA;FGF1;FGF2;MET
3	growth factor activity (GO:0008083)	1,24E-03	22,771	9/69	EPGN;LIF;PDGFA;INHBA;FGF1;NGF;FGF2;DKK1;PGF
4	phosphatidylinositol bisphosphate kinase activity (GO:0052813)	1,52E-03	21,451	9/71	FGF5;KITLG;ERBB3;KIT;PIK3CD;PDGFA;FGF1;FGF2;MET
5	transmembrane receptor protein tyrosine kinase activity (GO:0004714)	2,20E-03	20,923	8/61	DDR1;ERBB3;AXL;KIT;CRIM1;TEK;MET;FGFRL1
6	cadherin binding (GO:0045296)	2,31E-03	11,633	23/313	PTPN1;ANXA2;HSPA5;TRPC4;ANK3;RANGAP1;EHD1;SLC9A3R2;CDH4;MYO1B;EHD4;GPRC5A;KRT18;DIAPH3;MPRIP;CDH2;FLNB;ITGA6;PKP3;EZR;MB21D2;DBN1;CDK5R1
7	activin binding (GO:0048185)	5,51E-03	40,690	3/10	FST;FSTL3;ENG
8	transforming growth factor beta receptor binding (GO:0005160)	6,36E-03	17,987	6/44	BAMBI;INHBA;GDF6;SMAD6;BMP6;ENG
9	fibroblast growth factor receptor binding (GO:0005104)	9,54E-03	30,331	3/12	FGF5;FGF1;FGF2
10	growth factor receptor binding (GO:0070851)	2,49E-02	8,374	8/92	FGF5;EPGN;PDGFA;PTPRJ;ITGA5;FGF1;FGF2;PGF
