# Supplementary material for: Cell Sheets from Adipose Tissue MSC Induce Healing of Pressure Ulcer and Prevent Fibrosis via Trigger Effects on Granulation Tissue Growth and Vascularization
Source: Int J Mol Sci. 2020 Aug 4;21(15):5567. doi: 10.3390/ijms21155567 (PMC7432086; doi:10.3390/ijms21155567)
Supplement: Supplementary file 1 [file ijms-21-05567-s001.zip › supplementary/Supplementary R2/SupplementM1 (14-21d).pdf]

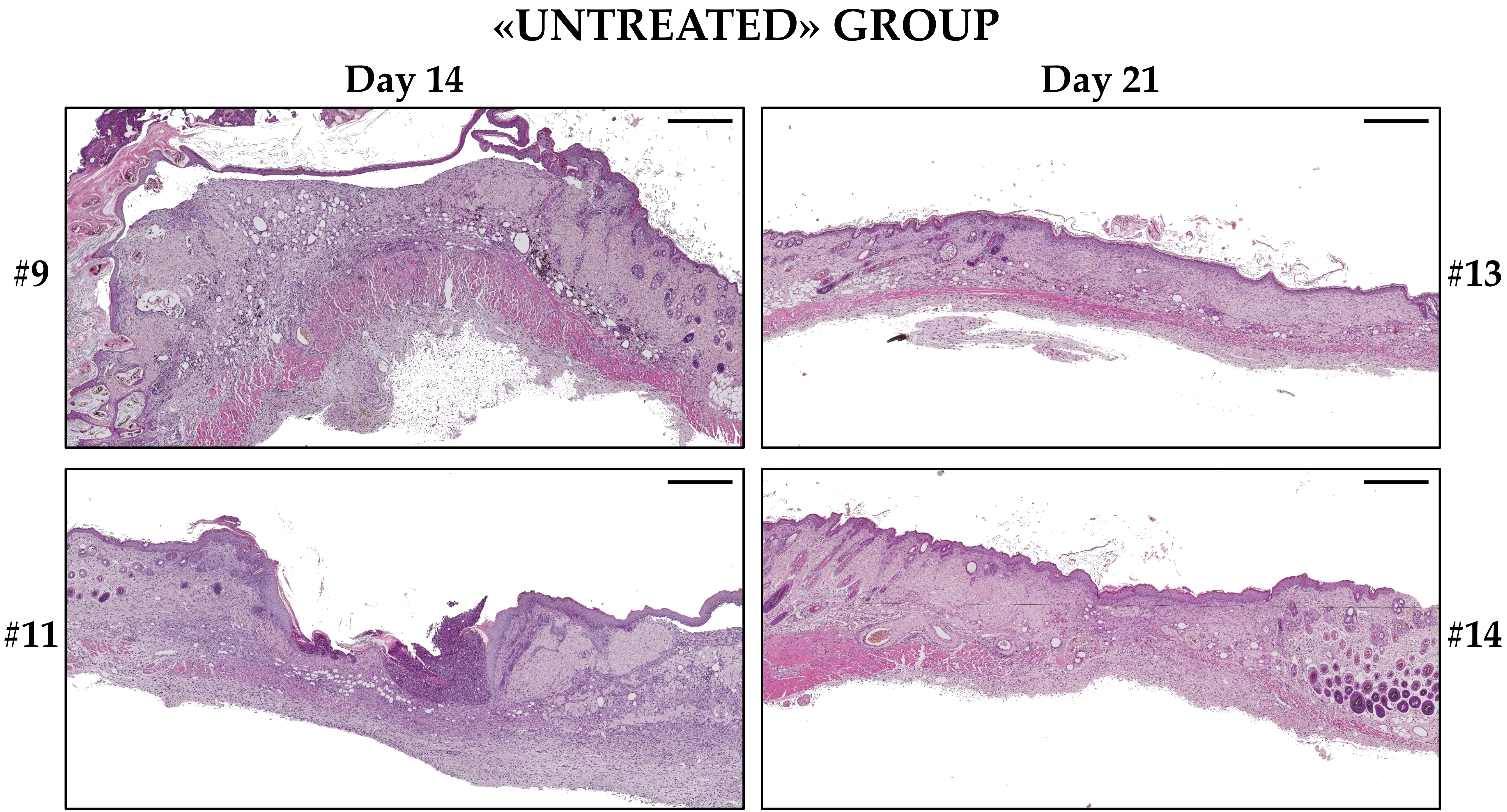
«UNTREATED» GROUP
Day 14
Day 21
#9
#13
#11
#14

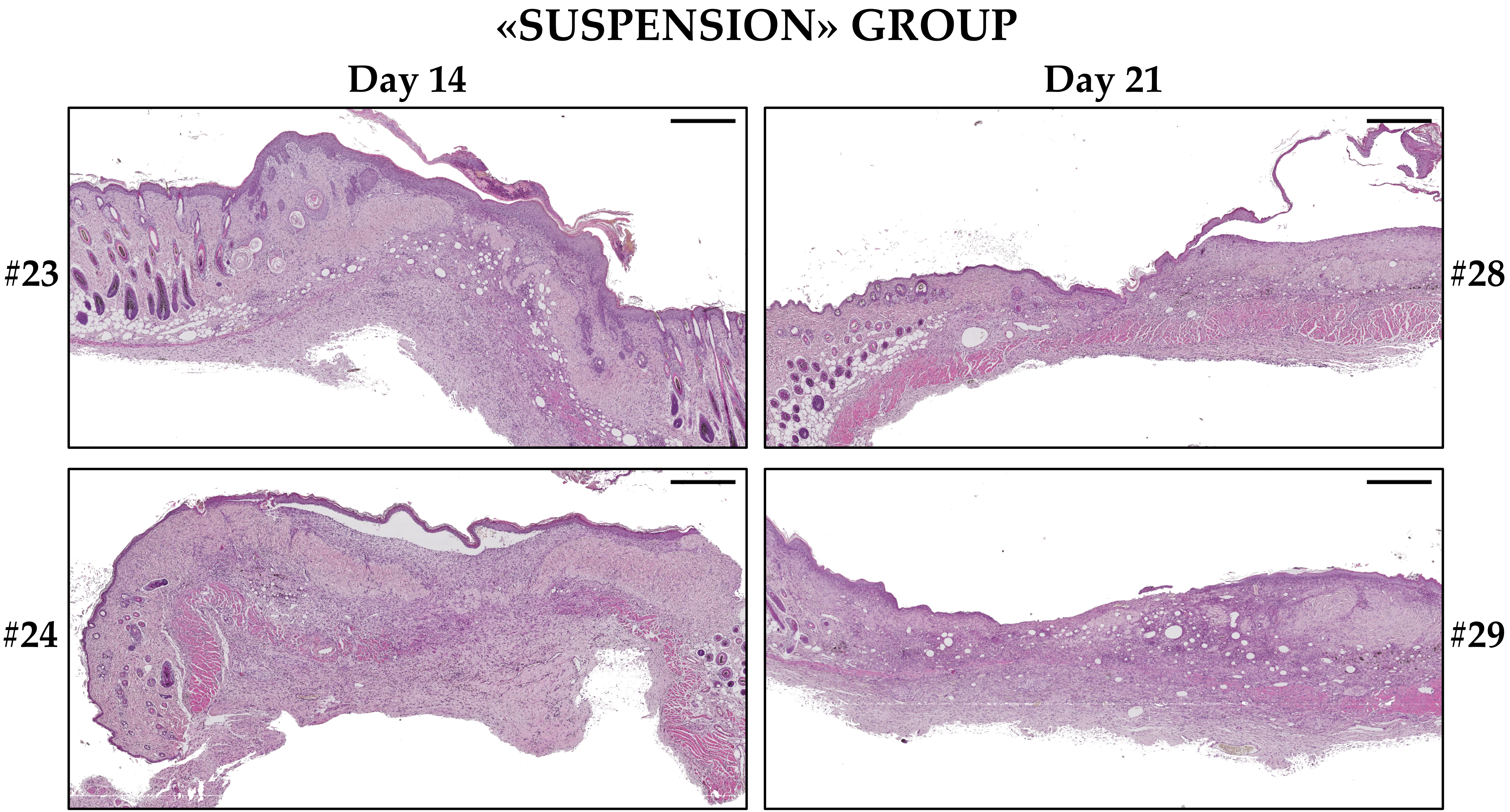
«SUSPENSION» GROUP
Day 14
Day 21
#23
#28
#24
#29

«SECRETOME» GROUP

Day 14 | Day 21

#55 | #58

#57 | #60

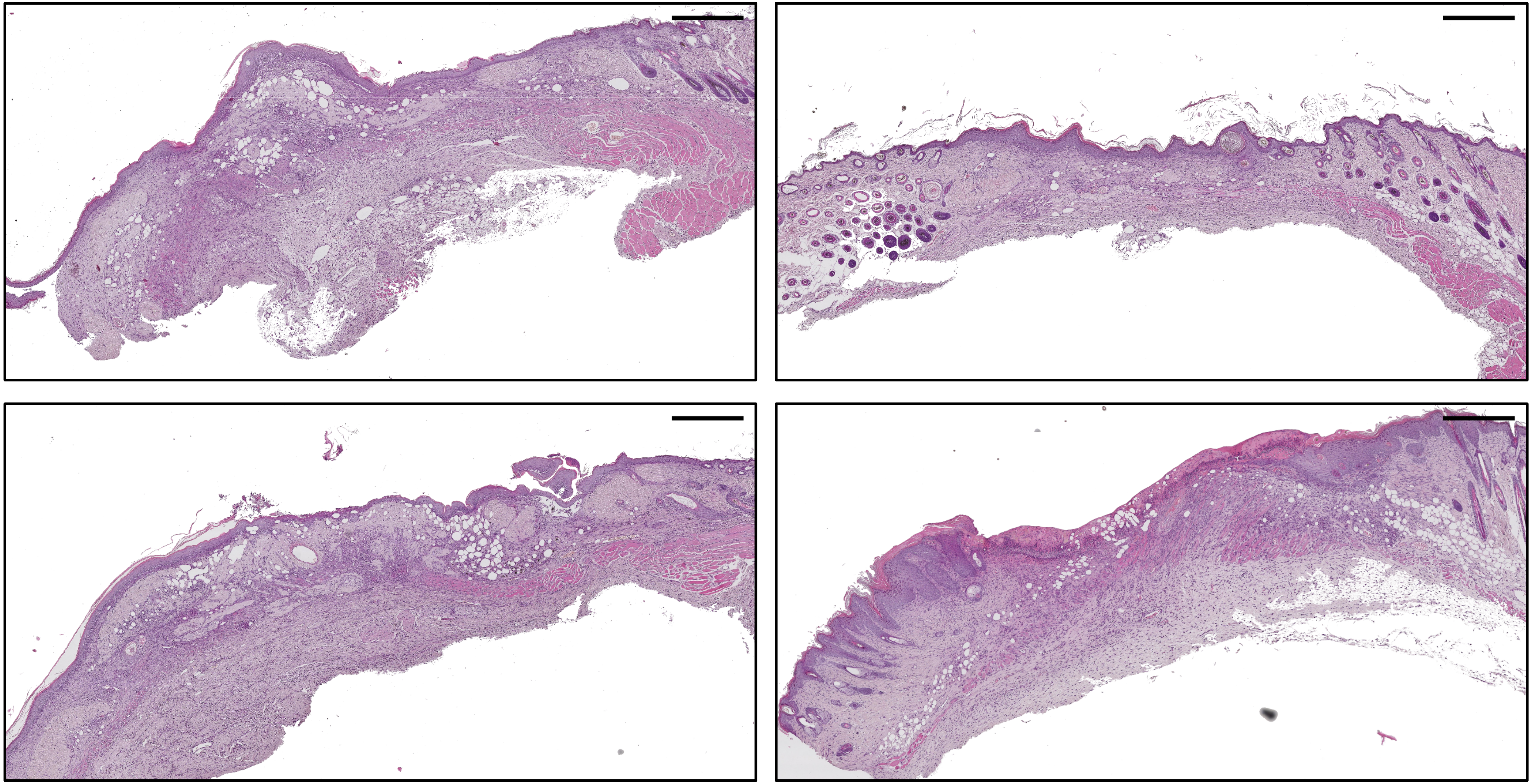

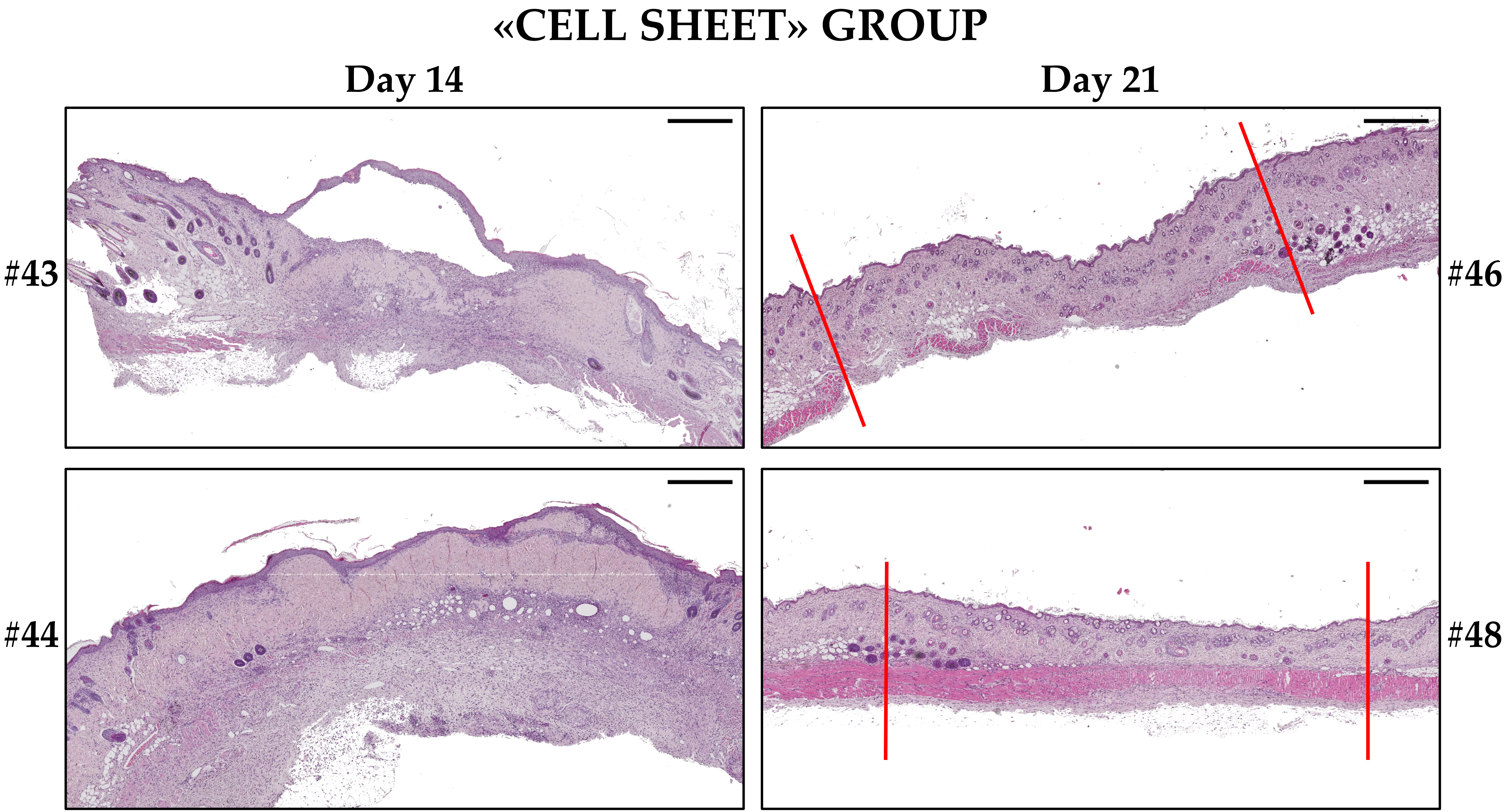
«CELL SHEET» GROUP
Day 14
Day 21
#43
#46
#44
#48
