# Supplementary figures and images for: Cell Sheets from Adipose Tissue MSC Induce Healing of Pressure Ulcer and Prevent Fibrosis via Trigger Effects on Granulation Tissue Growth and Vascularization
Source: Int J Mol Sci. 2020 Aug 4;21(15):5567. doi: 10.3390/ijms21155567 (PMC7432086; doi:10.3390/ijms21155567)

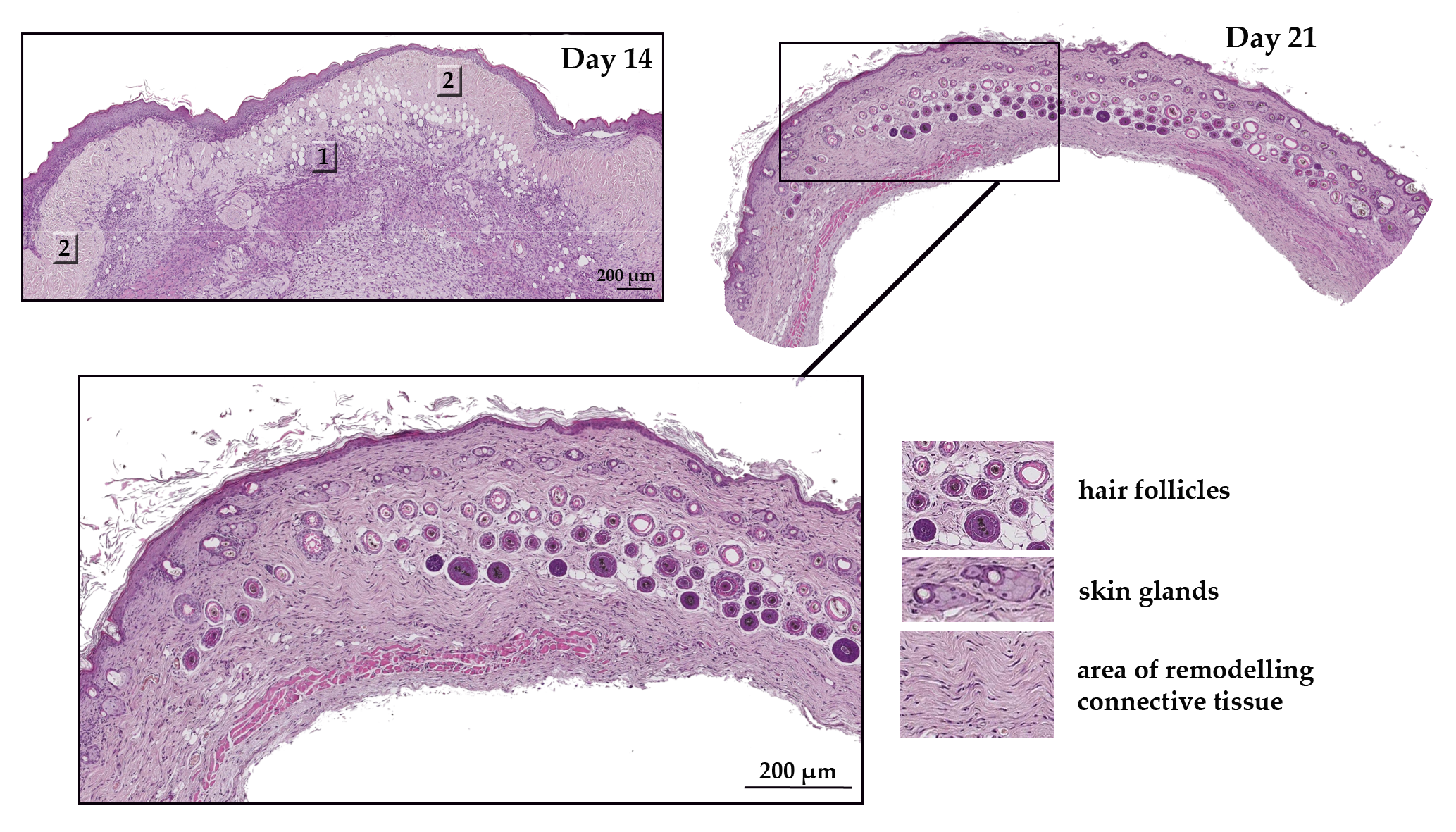

Supplement: Supplementary file 1 [file ijms-21-05567-s001.zip › supplementary/Supplementary R2/SupplementS1_Histological assessment of healing process of pressure ulcer after transplantation of Cell Sheet from MSC.tif]

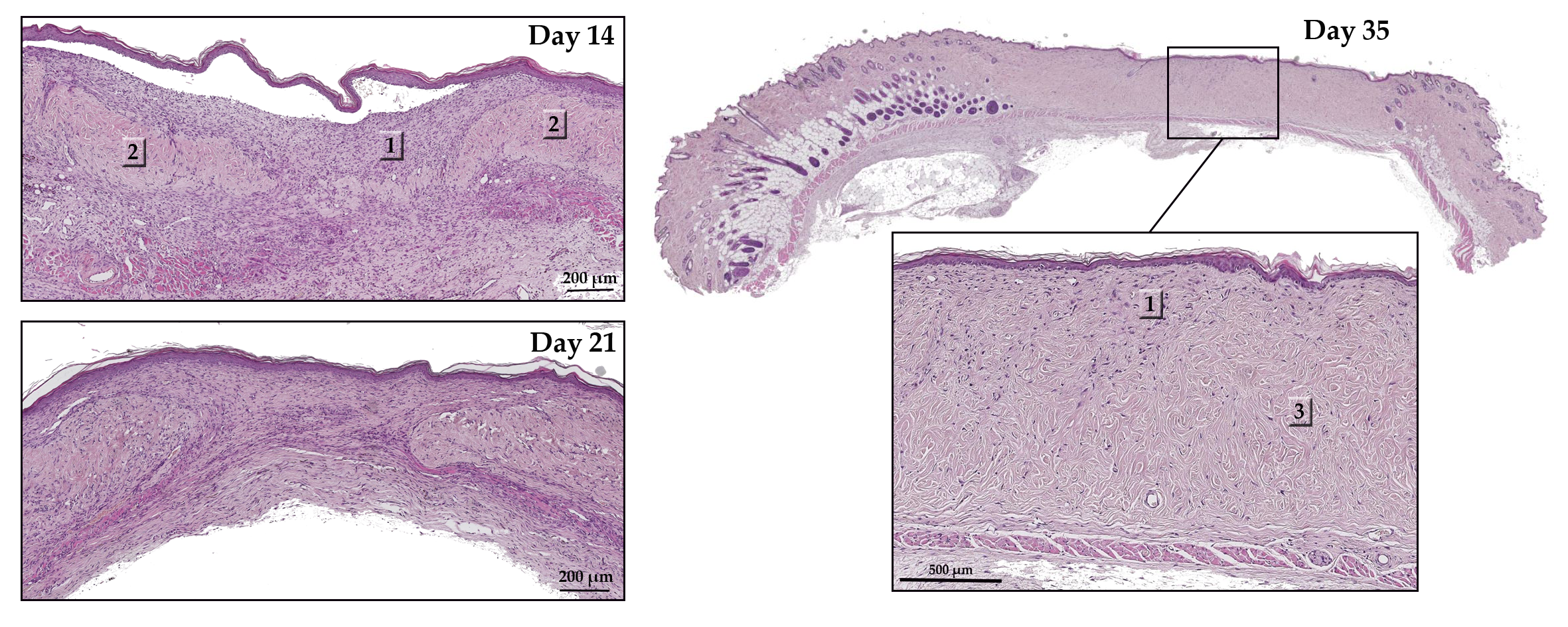

Supplement: Supplementary file 1 [file ijms-21-05567-s001.zip › supplementary/Supplementary R2/SupplementS2_Histological assessment of healing process of pressure ulcer in control animals.tif]

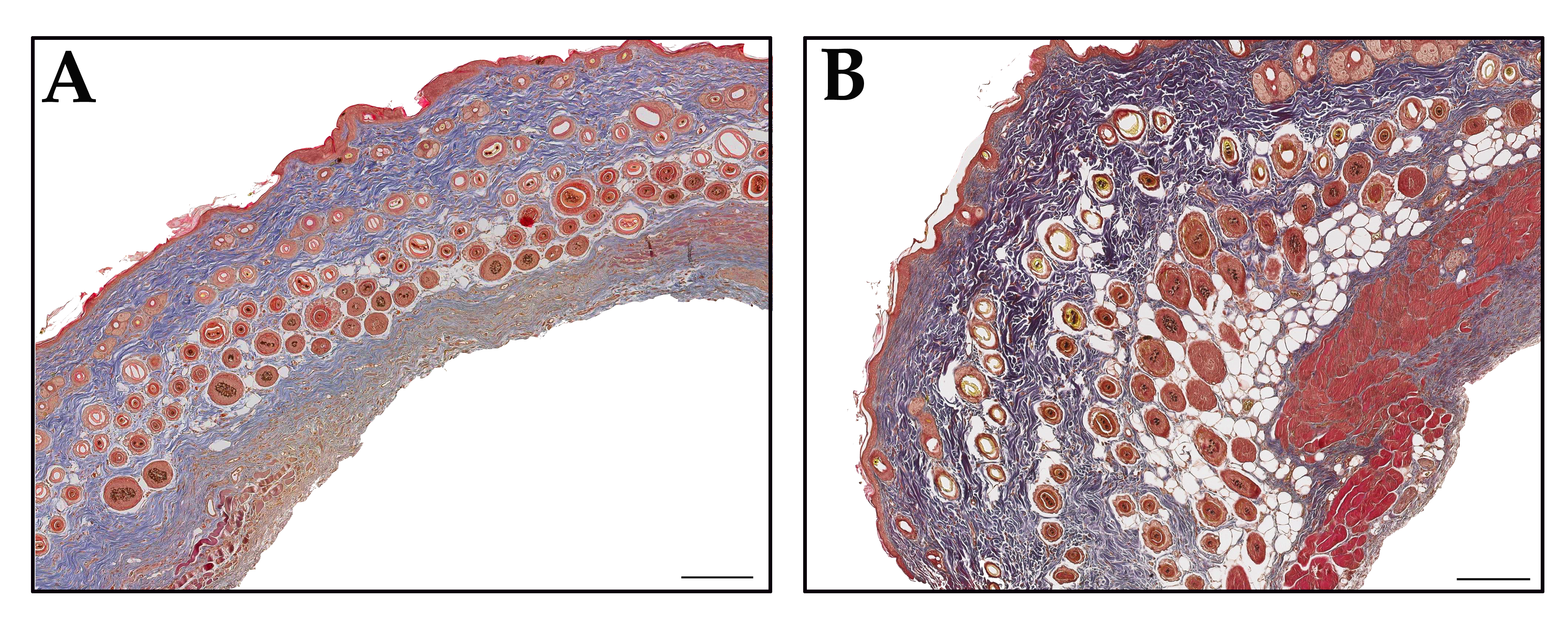

Supplement: Supplementary file 1 [file ijms-21-05567-s001.zip › supplementary/Supplementary R2/SupplementS3_Healthy-skin_masson.tif]
